# Standardization of laboratory bioassays for the study of Triatoma sordida susceptibility to pyrethroid insecticides

**DOI:** 10.1186/s13071-015-0726-4

**Published:** 2015-02-19

**Authors:** Grasielle Caldas D’Ávila Pessoa, Letícia Cavalari Pinheiro, Marcela Lencine Ferraz, Bernardino Vaz de Mello, Liléia Diotaiuti

**Affiliations:** Laboratório de Triatomíneos e Epidemiologia da Doença de Chagas, Centro de Pesquisa René Rachou, Av Augusto de Lima, 1715 Barro Preto, Belo Horizonte, MG CEP 30190-002 Brazil; Secretaria de Saúde do Estado de Minas Gerais, Rodovia Prefeito Américo Gianetti, s/n., 0 - Serra Verde, Belo Horizonte, MG CEP 31.630-901 Brazil

**Keywords:** Insecticide resistance, Bioassays, Triatominae, *Triatoma sordida*

## Abstract

**Background:**

Increasing reports of high-resistant Triatominae populations concerns scientists and sanitarians as little is known about the factors behind the occurrence of such phenotype and its real impact on vector control strategies. Moreover, the utilization of a large variety of methodologies hinder the comparison of the reported studies.

**Methods:**

This work aims to review laboratory bioassays, redefining the assessed biological features (age, generation and insecticide application area) and technical procedures (mortality recording time and the ideal diagnostic dose).

**Results:**

Results were not influenced by the insecticide application area in nymphs or by their generation. Three days-old specimen’s revealed lower susceptibility to the tested insecticide. We determined that it is more appropriate to record mortality 72 h after treatment with insecticide, as well as using a diagnostic dose of 1xDL_99_.

**Conclusion:**

This work suggests more adequate methodological parameters for assessing insecticide resistance in triatomines, which also allows the comparison of results obtained by different research groups. For laboratory bioassays, we recommend: 1) the use of first instar nymphs from first or second generation; 2) 3 day-old specimens; 2) application of insecticide in the dorsal or ventral abdomen area; 3) mortality recording 72 h after treatment with pyrethroids and 4) a diagnostic dose of 1x LD_99_.

## Background

Until recently, Triatominae insecticide resistance was considered sporadic with a small probability of occurring as the cycle of such insects is considerably long, presenting lower opportunities to select resistant individuals [1-6]. However, reports of resistant triatomine populations since the last decade have caused great impact among the scientific community, challenging them to find new alternatives for vector control strategies. In this case, insecticide resistance became the priority in the Chagas disease scenario, which requires better characterization and delimitation.

Currently, there is only one protocol guiding the studies of triatomine insecticide resistance, which belongs to the WHO [7]. However, the utilization of a large variety of methodologies has been reported [8-23], which prevents direct comparison of results and, in a more practical way, the comprehension of the real impact of those results in field vector control strategies.

According to the methodology proposed by the WHO [7], studies to assess the susceptibility of triatomines to insecticides in laboratory must be performed with first instar nymphs of the F1 generation (5 to 7 days of age, fasting, weight 1.2 ± 0.2 mg). Topical application of insecticide must be in the dorsal of abdomen and the mortality recorded at 72 h after treatment with the active chemical.

The choice of the susceptibility reference lineage – SRL is critical during resistance studies since it acts as the sensitivity standard for comparisons with field populations. In this context, PAHO [24] defined a SRL as a lineage with more than five generations in laboratory (without contact with insecticide and inclusion of external material), and/or one collected from an area never treated with insecticide.

Therefore, the purpose of this work was to reassess the different biological features and technical procedures used in previously reported insecticide resistance literature, comparing them to those proposed by the WHO [7].

## Methods

In Southeast Brazil, *Triatoma sordida* is the species that presents the greatest risk for vectorial transmission of *Trypanosoma cruzi* [25]. This species is endemic in Brazil. We used a susceptibility reference lineage derived from a peridomestic population captured in 1992 in Uberaba, Minas Gerais (19°44′52″S 47°55′55″O). Insecticide resistance was evaluated in four peridomestic populations from endemic areas of Minas Gerais, where the *Chagas Disease Control Program* (PCDCh) has been uninterrupted for the last 30 years: Monjolos (18°19′30″S 44°7′08′O), Buenópolis (17°52′22′S 44°10′48′O), Presidente Juscelino (18°38′13′S 44°03′28′O) and Coração de Jesus (16°41′06′S 44°21′54′O). In concordance with WHO [7], bioassays were performed with the aid of a Hamilton microsyringe equipped with a repeating dispenser. Topical applications of 0.2 μL of serial deltamethrin dilutions in acetone (0.01 to 9.0 ng/μL) were delivered onto the abdomen of first instar nymphs. At least ten insects (fasting – weight 1.2 ± 0.2 mg) were used per dose and per replicate. A minimum of six doses flanking the lethal dose 50% (LD_50_) causing 10% and 90% mortality, were used for each treatment. Each experiment was replicated at least three times. Control groups received only pure acetone. Treated and control insects were transferred to Petri dishes free of insecticide and kept in an environment under controlled temperature and humidity.

The mortality was recorded at 24 and 72 h. The criterion for mortality was based on the inability of the nymphs to walk from the center to the border of an 11-cm paper disc. Bioassay data were subjected to Probit analysis [26] to estimate the lethal dose (nanograms per nymph) that kills 50% of treated individuals (LD_50_). Resistance ratios 50% (RR_50_) and 95% Confidence Intervals (CI) for each population was calculated by comparing the dose response curves between the studied populations and the reference lineage.

Considering the initial proposal of this work, we assessed the following: 1) biological parameters - age (1, 3 and 5 days of life); specimens generations (F1 or F2); insecticide application area (dorsal or ventral of abdomen); and 2) technical procedures – mortality recording time (24 or 72 h); and ideal diagnostic dose in laboratory qualitative testing (1xLD_99_ or 2xLD_99_).

Results from the experiments were analyzed through non-parametric hypothesis tests considering the nature of the data (paired/independent samples and number of groups compared). For all the performed tests, differences between groups were considered significant when p-values were below or equal to α = 0.05.

## Results

For each of the five evaluated populations, the Kruskal-Wallis test performed on the 3 age groups generated a p-value = 0.045 to DL_50_. This means that a significant difference exists between at least 2 of the age groups. Thus, post-hoc tests (correcting for multiple comparisons [27]) were performed in order to show between which of the groups this difference existed. When comparing between the 1 and 3 days-old groups a significant difference was observed (p ≤ 0.05). The difference observed between the 3 and 5 days-old groups was significant as well (p ≤ 0.05). In contrast, the difference between the 1 and 5 days-old groups was not significant (p = 0.61). The statistical analyses thus indicated that for all the tested populations the 3-days-old nymphs showed less susceptibility to deltamethrin when compared by the age group parameter (Table 1).

**Table 1:** **Comparison of toxicity produced by deltamethrin applied to N1** ***T. sordida*** **of ages 1, 3 and 5 days (F1 generation); p = 0.045, Kruskal-Wallis test; p (1 day, 3 days) = 0.04, p (1 day, 5 days) = 0.61, p (3 days, 5 days) = 0.05, Kruskal-Wallis post-hoc tests**

**Populations**	**LD**_**50**_ **(CI 95%)**		
**Municipality (Locality)**	**1-day old**	**3-day old**	**5-day old**
Uberaba - SRL	0.062 (0.01 – 0.07)	0.300 (0.29 – 0.31)	0.065 (0.05 – 0.08)
Monjolos (Cipó)	0.126 (0.09 – 0.16)	0.417 (0.34 – 0.50)	0.173 (0.13 – 0.22)
Buenópolis (Cercado)	0.190 (0.17 – 0.26)	0.471 (0.42 – 0.56)	0.237 (0.19 – 0.30)
Presidente Juscelino (Mandioca)	0.301 (0.23 – 0.45)	0.598 (0.36 – 0.68)	0.360 (0.29 – 0.43)
Coração de Jesus (Barriguda)	0.412 (0.15 - 0.56)	0.616 (0.39 – 0.78)	0.444 (0.38 – 0.52)

Note: LD_50_: Lethal dose 50%; CI: Confidence Interval.

No significant differences were observed when comparing the toxicological profiles of two insect generations (p = 0.8, Mann-Whitney test) as shown in Table 2.

**Table 2:** **Comparison of toxicity produced by deltamethrin applied to N1** ***T. sordida*** **of generations F1 and F2 (5-days old); p = 0.84, Mann-Whitney test**

**Populations**	**LD**_**50**_ **(CI 95%)**	
**Municipality (Locality)**	**F1 generation**	**F2 generation**
Uberaba – SRL	0.016 (0.05 – 0.021)	0.065 (0.05 – 0.08)
Monjolos (Cipó)	0.151 (0.06 – 0.25)	0.173 (0.13 – 0.22)
Buenópolis (Cercado)	0,199 (0.06 – 0.36)	0.237 (0.19 – 0.30)
Presidente Juscelino (Mandioca)	0.410 (0.31 – 0.51)	0.360 (0.29 – 0.43)
Coração de Jesus (Barriguda)	0.415 (0.26 – 0.45)	0.444 (0.38 – 0.52)

Note: LD_50_: Lethal dose 50%; CI: Confidence Interval.

As shown in Table 3, when assessing the effect of insecticide application onto different abdominal areas, no significant differences were found when comparing dorsal and ventral surface applications (p = 0.4, Mann-Whitney test).

**Table 3:** **Comparison of toxicity produced by deltamethrin applied on dorsal or ventral abdomen of N1** ***T. sordida*** **(5-days old, F1 generation); p = 0.42, Mann-Whitney test**

**Populations**	**LD**_**50**_ **(CI 95%)**	
**Municipality (Locality)**	**Dorsal abdomen**	**Ventral abdomen**
Uberaba – LRS	0.065 (0.05 – 0.08)	0.623 (0.36 – 0.69)
Monjolos (Cipó)	0.173 (0.13 – 0.22)	0.172 (0.14 – 0.19)
Buenópolis (Cercado)	0.237 (0.19 – 0.30)	0.226 (0.18 – 0.29)
Presidente Juscelino (Mandioca)	0.360 (0.29 – 0.43)	0.360 (0.30 – 0.39)
Coração de Jesus (Barriguda)	0.444 (0.38 – 0.52)	0.425 (0.32 – 0.45)

Note: LD_50_: Lethal dose 50%; CI: Confidence Interval.

Regarding the parameter of time of death recordings (Table 4), significant differences were observed when comparing the amount of dead triatomines registered at 24 h and 72 h (p ≤ 0.05, Wilcoxon test).

**Table 4:** **Mortality of N1** ***T. sordida*** **(5-days old, F1 generation), in response to the 1xLD**_**99**_ **of SRL, carried out 24 and 72 h pos-treatment; p=0.006, Wilcoxon test**

**Populations**	**RR**_**50**_ *	**Mortality**	
**Municipality (Locality)**		**24 hours**	**72 hours**
Uberaba - SRL	1.0	30	29
Monjolos (Cipó)	2.6	30	28
Buenópolis (Cercado)	3.6	24	16
Presidente Juscelino (Mandioca)	5.5	25	17
Coração de Jesus (Barriguda)	6.8	25	11

Note: RR_50_*: 50% resistance ratio (data to Pessoa [27]).

Finally, Table 5 presents the mortality recorded when assessing the effect of the amount of insecticide applied: 1xLD_99_ and 2xLD_99_. When applying 1xLD_99_, different mortality rates were recorded for the tested populations, which present different toxicological profiles. The use of 2xLD_99_ produced 100% mortality rate in the same populations.

**Table 5:** **Mortality of N1** ***T. sordida*** **(5-days old, F1 generation), in response to the 1xLD**_**99**_ **and 2xLD**_**99**_ **of SRL, carried out 72 h pos-treatment**

**Populations**	**RR**_**50**_ *	**DD = 1xLD**_**99**_	**DD = 2xLD**_**99**_
		**(0.4375 a.i. ng/nymph)**	**(0.875 a.i. ng/nymph)**
**Municipality (Locality)**		**Mortality (%)**	**Mortality (%)**
Monjolos (Cipó)	2.6	93.4	100
Buenópolis (Cercado)	3.6	53.4	100
Presidente Juscelino (Mandioca)	5.5	56.7	100
Coração de Jesus (Barriguda)	6.8	36.7	100

Note: RR_50_*: 50% resistance ratio (data to Pessoa [27]); DD: Diagnostic dose; LD_99_: 99% Lethal dose.

## Discussion

The standardization of methodologies for studying insecticide resistance is a fundamental process for the implementation of routines to monitor triatominic populations, and it must therefore be considered strategic to vectorial control programs. Attending this objective, the “II Reunion Tecnica lationamericana monitoreo of resistance to insecticides in triatominos Chagas vectors” was held in 2005 in Panama. However, even after this meeting, a wide variety of methodologies are still in use, making it impossible to compare results directly and to comprise the real impact of these results on the effectiveness of the strategies used in the field.

Bioassays assessing insecticide susceptibility in nymphs of different ages revealed that 3 day-old triatomines are less susceptible than insects from 1-day old and 5-days old. 3 day-old nymphs differ from 1 day-old nymphs probably due to the presence of a fully formed exoskeleton and also differ from 5 day-old nymphs, as the latter still have stored energy from the egg stage that can be allocated for detoxification of insecticide. When comparing the insecticide activity of 1-dodecanol on the development of the cuticle in *R. prolixus and T. infestans,* was observed that recently hatched first instar nymphs (1–3 hours old) are more vulnerable than older nymphs (24–36 hours old) [28]. Pedrini et al. [29] demonstrated through electronic microscopy that Argentinean populations of *T. infestans,* resistant to deltamethrin, presented a thicker exoskeleton (32.1 ± 5.9 μm) than susceptible populations (17.8 ± 5.4 μm). These studies indicate that the cuticle is an important variable in the intoxication of bugs as it is the first barrier blocking insecticides from hitting its target site. Considering that the great majority of tests use pools formed by nymphs of 1 to 7 days of age this information is important considering different responses considering the lifetime.

No differences were found using first or second generation of insects. The selection of resistance genes is expected to occur only under direct pressure with active chemicals [30]. Therefore, it is assumed that loss of resistance genes in insects collected from the field kept in insectary would be a slow process or even absent in the population due to the long lifecycle of most Triatominae species, therefore not interfering in the results obtained. Furthermore, the possible use of second generation of insects useful as the number of insects collected in field is usually reduced of triatomines, in small groups, which makes it difficult, or even impossible, to generate the sufficient specimen numbers for the conclusion of bioassays.

Although WHO [7] recommend the topical application of insectide at the dorsal part of the abdomen in insects, some works suggest instead a ventral application [10,31]. In our tests, the location where the insecticide was applied did not affect mortality rate. In routine these results are useful once affected by the acetone odor, the specimens tend to expose the ventral part of the abdomen in order to allow more ventilation into their spiracles and this behavior forced the researcher to untap them and then to proceed with the application of the insecticide. Besides, it was observed also that the greater the RR_50_, the greater the knockdown recovery.

Regarding the time required for the mortality reading, the protocol proposed by WHO [7] recommends the reading to be performed 72 h after treatment with active chemicals. However, many studies have presented such readings after 24 hours [10,11,13,15,17,18,20-23] disregarding the knockdown effect – a momentary paralysis followed or not by full motion recovery caused by pyrethroid insecticide [32]. Our studies corroborate with what is preconized by the WHO as we verified the recovery from the knockdown effect only 72 hours after treatment with insecticide. Supporting the WHO protocol, we observed that many insects paralyzed at 24hrs end up recovering after 72h. Therefore, it is possible that its occurring erroneous record of dead insects with those under knockdown that are subject to recovery, underestimating previously found lethal doses and derived resistance ratios.

Diagnostic doses is a tool that allows the distinction of the field population vigor against the SRL. In studies with triatomines, in spite of what is preconized by the WHO [7] concerning the use of 1xLD_99_, some authors have adopted 2xLD_99_ [17,33] as recommended for mosquitoes [34]. The aim of DD is to indicate possible field population with insecticide resistance, killing the most susceptible specimens and the minimal resistant specimens. The use of 1xLD_99_ was considered more appropriate, which also allowed us to establish a positive correlation between the obtained mortality and the different toxicological profiles indicated by the resistance ratios (RR). On the other hand, results with 2xLD_99_ invariably generate a mortality of 100%.

We emphasize the issue of the sampling number and we recommend the utilization of 10 nymphs in each testing, including triplicate [7]. In this context, Amelloti et al. [9] demonstrated that a single female, kept isolated throughout its lifecycle, has the potential to generate more resistant offspring when young and, as it ages, they generate more susceptible descendants. It points to the complexity of genetic variability at an individual level, raising questions on the impact of such variation in a populational context.

## Conclusion

In conclusion, we recommend for the laboratory bioassays of triatomines the following: 1) use of first instar 3 day-old nymphs from first or second generation 2) application of insecticide either at the dorsal or the ventral abdomen; 3) measurement of recovery at 72 h after treatment with pyrethroid and 5) the use of diagnostic dose on 1x LD_99_.
